# The impact of climate change on heat-related mortality in six major cities, South Korea, under representative concentration pathways (RCPs)

**DOI:** 10.3389/fenvs.2014.00003

**Published:** 2014-02-19

**Authors:** Young-Min Kim, Soyeon Kim, Yang Liu

**Affiliations:** 1 Department of Environmental Health, Rollins School of Public Health, Emory University, Atlanta, GA, USA; 2 Department of Social and Preventive Medicine, Sungkyunkwan University School of Medicine, Suwon, Korea

**Keywords:** climate change, heat-related mortality, relative risk, generalized linear Poisson model, representative concentration pathway (RCP)

## Abstract

**Background:**

We aimed to quantify the excess mortality associated with increased temperature due to climate change in six major Korean cities under Representative Concentration Pathways (RCPs) which are new emission scenarios designed for the fifth assessment report of the Intergovernmental Panel on Climate Change (IPCC).

**Methods:**

We first examined the association between daily mean temperature and mortality in each during the summertime (June to September) from 2001 to 2008. This was done using a generalized linear Poisson model with adjustment for a long-term time trend, relative humidity, air pollutants, and day of the week. We then computed heat-related mortality attributable to future climate change using estimated mortality risks, projected future populations, and temperature increments for both future years 2041–2070 and 2071–2100 under RCP 4.5 and 8.5. We considered effects from added days with high temperatures over thresholds and shifted effects from high to higher temperature.

**Results:**

Estimated excess all-cause mortalities for six cities in Korea ranged from 500 (95% CI: 313–703) for 2041–2070 to 2,320 (95% CI: 1430–3281) deaths per year for 2071–2100 under two RCPs. Excess cardiovascular mortality was estimated to range from 192 (95% CI: 41–351) to 896 (95% CI: 185–1694) deaths per year, covering about 38.5% of all-cause excess mortality. Increased rates of heat-related mortality were higher in cities located at relatively lower latitude than cities with higher latitude. Estimated excess mortality under RCP 8.5, a fossil fuel-intensive emission scenario, was more than twice as high compared with RCP 4.5, low to medium emission scenario.

**Conclusions:**

Excess mortality due to climate change is expected to be profound in the future showing spatial variation. Efforts to mitigate climate change can cause substantial health benefits via reducing heat-related mortality.

## INTRODUCTION

Studies worldwide have established a robust relationship between high temperature and excess mortality (Braga et al., 2002; Curriero et al., 2002; Gouveia et al., 2003; Anderson and Bell, 2009, 2011; Hajat and Kosatky, 2010; Basu and Malig, 2011; Gasparrini and Armstrong, 2011; Kim et al., 2011; Son et al., 2012). As an increase in the frequency and intensity of extreme hot weather is predicted in the future [Intergovernmental Panel on Climate Change (IPCC), 2012], its potential health impact raises a growing public health concern. A better understanding of the extent of their subsequent impact under climate change can help policy makers establish more effective adaptation strategies (e.g., improvement of the heat warning system in response to future heat waves) (O’Neill and Ebi, 2009). However, few studies have quantitatively estimated the heat-related health impacts attributable to future climate change. A limited number of studies have projected the health impacts of future climate change via heat wave (Peng et al., 2011), temperature increase (Dessai, 2003; Knowlton et al., 2007), and air pollution (Bell et al., 2007; Tagaris et al., 2009; Post et al., 2012). Peng et al. (2011) reported that Chicago could experience between 166 and 2217 excess deaths per year attributable to heat wave in 2081–2100 based on three different climate change scenarios. Similarly, a study of heat-related mortality in Lisbon found that the potential increase of annual heat-related mortality rate ranged from 7.3 to 35.6 per 100,000 persons by the 2050s (Dessai, 2003).

Because the association between hot weather and health vary substantially in space (Curriero et al., 2002; Baccini et al., 2008; Hajat and Kosatky, 2010), studies of the health impact of future climate change need to capture the spatial heterogeneity in the health effects across geographical regions. To date, no study has investigated heat-related mortality under future climate change in South Korea.

Gasparrini and Armstrong showed that the “main effect” of heat waves on excess mortality due to independent effects of daily high temperatures can be greater than the estimated “added effect” due to sustained duration of heat (Gasparrini and Armstrong, 2011). This implies that most of the excess mortality due to heat waves can be estimated by the temperature effects of individual hot days. Non-linear relations between daily temperature and mortality (reverse J-shaped) have been observed; mortality risk decreases as temperature increases from the coldest days. After a certain critical temperature threshold, mortality risk increases as temperature increases (Curriero et al., 2002). An important question is how to assess the temperature change between the present and future while considering not only the days over the empirical temperature threshold, but also days with shifted hot-to-hotter temperature. To assess the potential impact of climate change on heat-related mortality, we adopted a new approach to include most “main effects” of over-threshold temperature, which can cause premature mortality, and the effects of extremely hot days, separately.

We aimed at quantifying the excess mortality associated with high temperatures due to future climate change in six major Korean cities based on the Representative Climate Pathways (RCPs), adopted for the fifth assessment report (AR5) of the Intergovernmental Panel on Climate Change (IPCC) (Moss et al., 2010; Rogelj et al., 2012). We first estimated heat-related mortality risks for these six cities using historical mortality and weather data, and subsequently calculated summertime temperature increase in the future using projected temperature based on a dynamical downscaling approach. Finally, we assessed the excess mortality due to climate change using future populations, the increased temperature, and the estimated high temperature related mortality risks.

## MATERIALS AND METHODS

### STUDY AREA

**FIGURE 1 ∣:**
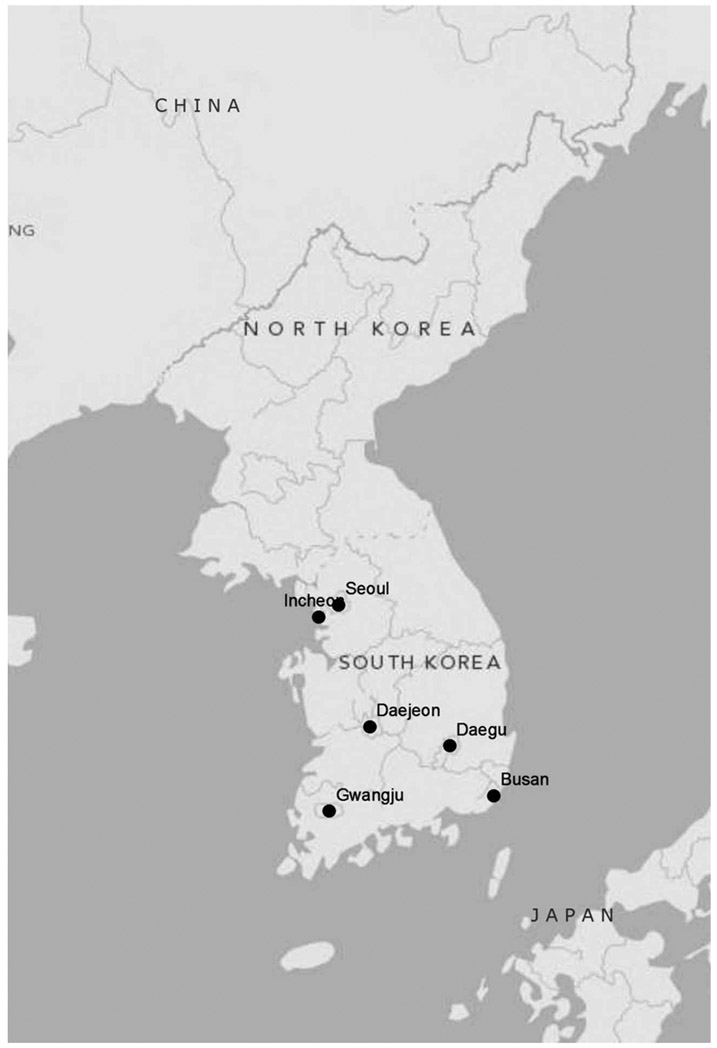
Location of six major cities in South Korea included in this study.

South Korea is located in the temperate region and has a hot and humid summer. The surface air temperature in South Korea has significantly increased by about 1.5°C during the past 100 years, which is greater than the global 0.74°C average increase (National Institute of Environmental Research, Ministry of Environment, Korea., 2010). Additionally, a previous study about future climate in Korea (Boo et al., 2005) reported that daily mean temperatures over Korea would increase by about 5.5°C between 1971–2000 and 2071–2100, whereas cold events would be rarer and less severe. We analyzed the effect of future climate change on heat-related mortality in six major cities including Seoul, Incheon, Daejeon, Daegu, Gwangju, and Busan. There are a total of 21 million residents in these cities, approximately half the national population of South Korea. Figure 1 presents the location of the six cities.

### HIGH TEMPERATURE AND MORTALITY RISK

#### Data

We used the following three types of data to estimate the present-day relationship between high temperature and mortality: meteorological records, air pollution, and daily death counts. Daily meteorological parameters, including temperature, and relative humidity, were obtained from the Korean Meteorological Administration (KMA) for six cities. Air pollution data were provided by the National Institute of Environmental Research, which monitors the ambient air pollution in South Korea. Daily mean concentrations of PM_10_ and ozone were first calculated for each monitoring site using hourly data and then the daily data were averaged by city. Mortality data were provided by the Statistics Korea. These records include date, residence, cause of death, and other demographic factors (e.g., age and sex). Based on the International Classification of Diseases Revision 10 (ICD 10) of primary or secondary disease codes, death counts for cardiovascular mortality (CVM) (ICD-10: I00-I99) and respiratory mortality (ICD-10: J00-99), and all-cause mortality were extracted for the six cities. Deaths attributable to external causes (ICD-10: V01-Y89) were excluded from the all-cause mortality. We used data only for the summertime (June 1 to September 30) of 2001–2008 to focus on the effect of high temperature. Summertime corresponds to the heat health warning system period as determined by the KMA.

#### Statistical analysis

We assessed the effect of high temperature on mortality using a Poisson generalized linear regression model (GLM) with natural cubic splines allowing over-dispersion (McCullagh and Nelder, 1989). Before fitting the GLM model, we used penalized regression curves of a generalized additive model (GAM) to examine the form of the relationship between temperature and mortality on the same day for the summertime and to determine thresholds of temperature (Wood and Augustin, 2002). When we fitted the GAM, confounders including relative humidity, air pollutants, day of week, and long-term time trend were controlled. During hot days, ozone can be produced more easily and the daily deaths increase as the ozone level increases (Rainham and Smoyer-Tomic, 2003; Ren et al., 2006, 2008). Particulate matter also can cause slight changes in estimates of coefficients from the model for heat-related mortality (Pattenden et al., 2003). Therefore, we controlled for ambient ozone and PM_10_ as confounding factors. In the GAM model, we allowed the data to determine the degree of smoothing.

**FIGURE 2 ∣:**
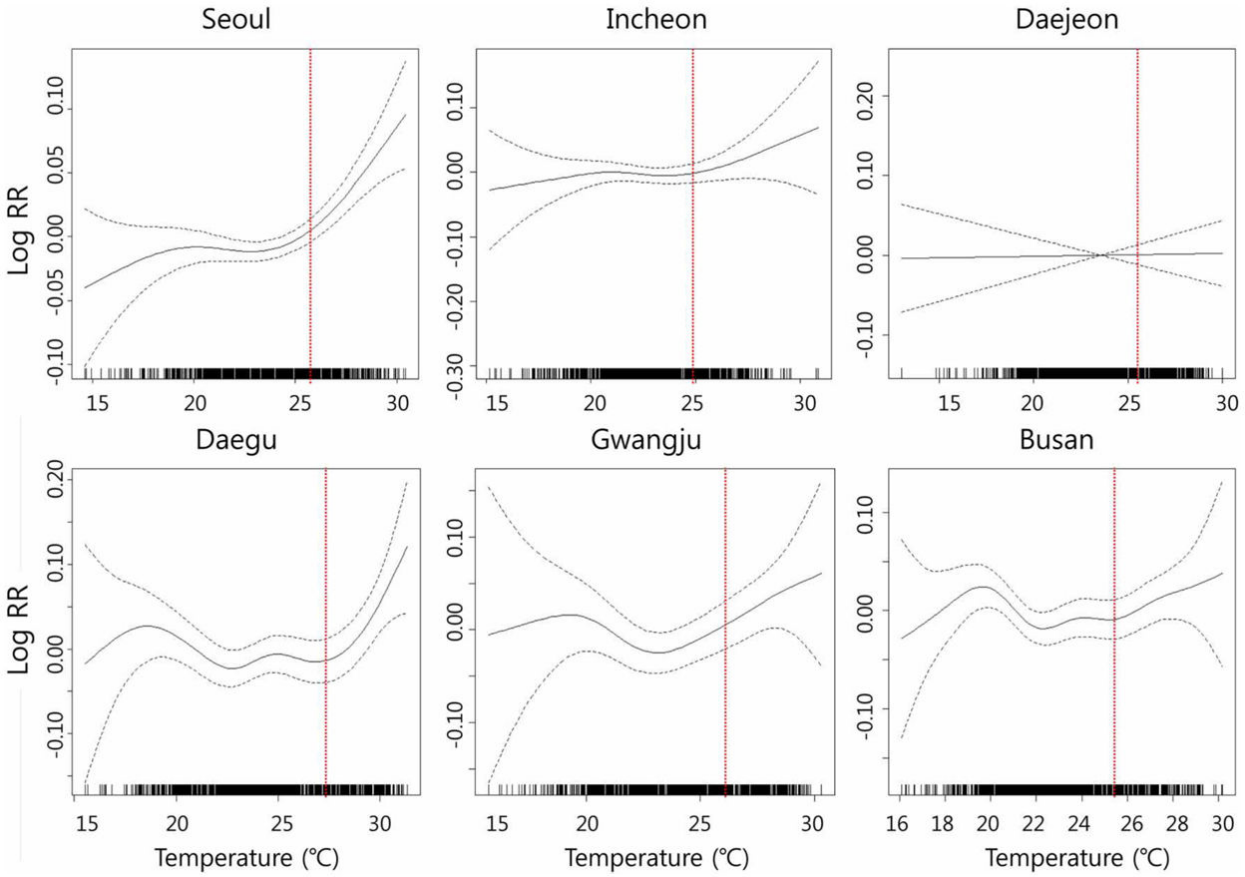
Penalized regression splines for all-cause mortality on daily mean temperature for summer (June to September in 2001–2008). Each figure shows the spline curve (the solid line) with a 95% confidence interval (dashed line); In each graph, X-axis indicates temperature (°C) and Y-axis indicates temperature-mortality relative risk (RR); Vertical dotted lines indicate 75th percentile of temperature for each city.

There is no commonly accepted threshold temperature to analyze the impact of high temperature on health. Different regions are likely to have different thresholds due to various demographics and adaptation practices. For example, Honda et al. (2007) suggested that a daily maximum temperature between the 80th and 85th percentile value of maximum temperature is the best parameter in Japan without any outlier city. We selected the upper 25% of temperature based on the GAM-fitted penalized regression curves since linear relationships between temperature and mortality are shown over the 75th percentiles of daily mean temperature (*T_q_^c^*) for all six cities (Figure 2). We then fitted the GLM only for the upper 25% of temperature datasets to quantify the temperature effects on mortality in each city. In addition, we tried to include as many days with adverse health effect of high temperature as possible by selecting the 75th percentiles of summertime daily mean temperature as shown in Figure 2.

After determining the threshold based on the GAM model, we fitted GLM model controlling for the same set of confounders in the GAM model. For the lag effect analysis, single-day lagged effect of 0 days (same day), 1 day (previous day), 2 days (2 previous days), and 3 days (3 previous days) in each city were applied. The model specifications are as follows:

(1) $$ln\left(E(Y_{t}^{c})\right)=\alpha +\beta^{c}T_{t-\text{lag}}^{c}+\gamma^{c}{\text{DOW}}_{t}+{\text{ns(Time}}_{t})+\text{ns}(RH_{t}^{c})\;\;+\text{ns}(AP_{it}^{c})$$

where *E*(*Y*_*t*_^*c*^) is the expected daily death count for city *c* on day *t*; α is the model intercept; β^*c*^ is the coefficient (slope) for the daily mean temperature for city *c* for a specific lag from day *t* (*T*_t−lag_^*c*^); γ^*c*^ is the vector of regression coefficients for day of week for city *c*; DOW_*t*_ is a 7-level indicator for day of week; *ns*(Time_*t*_) is the natural cubic spline of a variable representing time to adjust for long-term trends with 2° of freedom for each summertime (16° of freedom for 8 summers); *ns*(*RH*_*t*_^*c*^) is the natural cubic spline of relative humidity of city *c* on day *t* with 4° of freedom; and *ns*(*AP*_*it*_^*c*^) denotes natural cubic splines of air pollutants *i* (daily mean PM_10_ and ozone) for city *c* on day *t* with 4° of freedom.

After estimating city-specific high temperature effects, we combined all the data to evaluate the overall effect (pooled effect of temperature on mortality across the six cities). We conducted meta-regression analyses using the estimates derived from the GLM. To take into account heterogeneity across cities, we applied random effect model by using restricted maximum-likelihood estimation (Harville, 1977; Viechtbauer, 2010). The percent change was derived from relative risks (RR) using the formula (RR-1) × 100. Here, RR indicates the change rate of expected death due to a 1°C increase in temperature. In addition to computing the effects of high temperature for all ages, we also estimated the effect on the elderly population (over 65 years of age).

All statistical analyses were performed in R 2.15.1 (The Comprehensive R Archive Network: http://cran.r-project.org) using the “mgcv” package (version 1.7-22) for city-specific effects and “metafor” package (version 1.6-0) for overall effects. All tests were two-sided, and an alpha level of less than 0.05 was considered significant.

### FUTURE TEMPERATURE

Projected daily mean temperature data for the Korean Peninsula from 2000 to 2100 is publicly available through the Climate Change Information Center (CCIC)-KMA database (CCIC-KMA, 2012). The estimated future weather is based on the RCPs.

Climate researchers from different disciplines coordinated by the IPCC have established a new coordinated parallel process for developing new scenarios, RCPs. This starts with four scenarios of future radiative forcing levels (the changes in the balance between incoming and outgoing radiation to the atmosphere caused by changes in atmospheric constituents, such as carbon dioxide) [20]. Four RCPs were produced based on a comprehensive literature review: one high pathway for in which radiative forcing reaches >8.5 W/m^2^ by the year 2100 and continues to rise for some amount of time (RCP 8.5); two intermediate “stabilization pathways” in which radiative forcing is stabilized at approximately 6 W/m^2^ (RCP 6.0) and 4.5 W/m^2^ (RCP 4.5) after 2100; and one pathway where radiative forcing peaks at approximately 3 W/m^2^ before 2100 and then declines (RCP 2.6) (Moss et al., 2008). RCP 8.5, 6.0, 4.5, and 2.6 assume approximately 1,370, 850, 650, and 490 ppm CO_2_ equivalent (CO_2_-eq) concentrations in 2100, respectively. The best estimate of CO_2_-eq concentration in 2005 for long-lived greenhouse gases (GHGs) only is about 455 ppm CO_2_-eq (Moss et al., 2010).

To project regional climate change, CCIC-KMA used a dynamic downscaling method from global climate change projection by a coupled atmosphere-ocean general circulation model, HadGEM2-AO, with about 135-km resolution under RCP 4.5 and 8.5. To evaluate the agreement between HadGEM2-AO-modeled temperature and observation, the CCIC-KMA checked geographical distribution of temperature for the period of 1979–1999. The model simulates presented climate well for the patterns of global and zonal temperature distribution. Details about the performance of the HadGEM2-AO are presented in Baek et al. (2013). Compared to 24 Coupled Model Intercomparison Project (CMIP) models, the HadGEM2-AO showed some improvements especially for India and East Asia regions where CMIP3 models generally show bad performances (Baek et al., 2013). For the preparation of national climate change scenario, the CCIC-KMA downscaled the regional climate projections for the years 2000–2100 over the Korean peninsula domain with 1-km resolution (National Institute of Meteorological Research, Korea, 2011).

We used daily mean temperature for 2001–2010 for baseline and 2041–2100 for future temperature to compute the increased high temperature. We divided 2040–2100 into two time periods, 2041–2070 and 2071–2100, and averaged the daily mean temperature for each time period to reduce the variation of the model simulation. When the simulated temperatures were compared with observed daily mean temperature for same period, 2001–2008, the coefficient of determinant and slope were 0.95 and 1.01, respectively (Figure S1).

### EXCESS MORTALITY DUE TO CLIMATE CHANGE

Excess mortality due to high temperature in the future was calculated employing health impact function, derived from the log-linear function in the GLM model. The health impact function has been used in the estimation of health impact of heat wave and climate change (Dessai, 2003; Peng et al., 2011; Post et al., 2012) and air pollution (Tagaris et al., 2009; Fann et al., 2012). Basic formula is as follows;

(2) $$\Delta y=y_{0}\left[e^{\beta \Delta T}-1\right]\times D$$

where Δ*y* is expected number of excess deaths during summertime; *y*_0_ is the expected daily number of death without climate change (the product of the baseline mortality incidence rate per day and the exposed population); β is coefficient of the relationship between mortality and high temperature (above threshold); Δ*T* is the increased temperature in the future due to climate change (future temperature—present temperature); *D* indicates number of exposed days to high temperature.

Future mortality incidences, baseline mortality incidences without climate change, were calculated using projected populations, which were published by Statistics Korea (2012), and current mortality rates for each city. Statistics Korea projected future populations from 2010 to 2040 by considering population growth, natural increase rate and migration based on 2010 Korean Census Survey. We used the projected population in 2040 for both 2041–2070 and 2071–2100 because there was no projected population for 2071–2100. We used current non-accidental, all-cause mortality and CVM rates for the year of 2008 to calculate future baseline mortality incidences.

To calculate temperature changes in the future, 2041–2070 and 2071–2100, compared with the present (2001–2010), we divided the future summer days into two categories based on temperature level, days with above and below 75th percentile of daily mean temperature of the future summer for each city. Days with above 75th percentile of future summer temperature were used to calculate the temperature shift effect from high (present-day) to higher temperature (in the future) (“shifted” effect). We first calculated differences between averaged daily mean temperatures of the upper 25% of present summer days and those of future summer days (future temperature-present temperature) to apply for Δ*T* in equation 2. We applied 30.5 days (25% of June through September) for the number of exposed days to high temperature (*D* in Equation 2) to compute the “shifted” temperature effect on mortality.

Among future summer days below 75th percentile of future summer temperature, we selected days with daily mean temperatures higher than *T_q_^c^*, the 75th percentile of present summer temperature by city, to evaluate the effect of added high temperature days due to climate change. We averaged the increased daily mean temperatures over *T_q_^c^* (Daily mean temperature of newly added day over *T_q_^c^* − *T_q_^c^*) by city and applied the averaged increase in temperature for Δ*T* in Equation 2. The average of the increase in temperature over *T_q_^c^* and the number of added days with temperature over *T_q_^c^* was used as Δ*T* and *D* in Equation 2 to estimate the impact of newly “added” high temperature over *T_q_^c^* (“added” effect). “Added days” in Table 3 indicates the number of exposed days with over *T_q_^c^*, *D*, used for the “added” effect. Figure S2 depicts how future summer days over present threshold, *T_q_^c^* are classified into “added” and “shifted” high temperature days.

Finally, we estimated excess mortality caused by climate change using the estimated mortality risks, projected future populations, and temperature increments. We first estimated excess mortality caused by “shifted” high temperatures (i.e., from high to higher temperature) and by newly “added” days over *T_q_^c^* in the future, separately. We then summed them to estimate the total effect of climate change. We computed excess mortality for each city using city-specific mortality risks and overall impact using the pooled mortality risk. The effects on respiratory mortality were not statistically significant in all cities, including the overall effect. Therefore, excess death counts for only all-cause mortality and CVM were estimated.

## RESULTS

### MORTALITY RISK

Table 1 shows summary statistics of study population, weather, and air pollutants of the summertime in the six cities. The population of the six cities ranged from 1,462,133 (Gwangju) to 10,081,017 (Seoul) for 2008. In 2040, the populations of four cities (Seoul, Daegu, Gwangju, and Busan) are expected to decrease slightly, while those of Incheon and Daejeon increase. Overall, the population of the six cities is predicted to decrease by about 500,000. Averaged daily death counts during the summertime for 2001–2008 ranged from 12.9 (Daejeon) to 88.5 (Seoul) for non-accidental all-cause mortality and from 3.1 (Gwangju) to 23.7 (Seoul) for CVM. Daily mean temperatures of 75th percentile of summertime were slightly higher in cities located at relatively lower latitude and inland area (e.g., Daegu and Gwangju) than cities at higher latitude and adjacent to the sea (e.g., Incheon). PM_10_ concentrations were on a similar level across the six cities, whereas daily mean ozone concentration in Daejeon (12.9 ppb) was much lower than other cities during the summertime.

Figure 2 depicts the smoothing plots of relative risk of temperature on all-cause mortality for the six cities during the summertime derived from GAM with adjustments for long-term time trend, relative humidity, and air pollutants. The relationship between daily mean temperature and mortality vary by city. Thresholds of all-cause mortality risks were found in relation to daily mean temperature during the study period except for Daejeon. Linear relationships between temperature and mortality are shown over the 75th percentiles of daily mean temperature for all six cities.

The estimated mortality risks on days with daily mean temperature greater than the 75th percentiles of summertime derived from GLM with natural cubic splines are presented in Table 2. Percent increases of all-cause mortality due to high temperature were the highest in same day models in most cities. The effect of high temperature on all-cause mortality was the highest in Daegu, showing 3.5% (95% CI: 0.4–6.7%) and 5.8% (95% CI: 1.6–10.1%) increase due to 1°C increase in daily mean temperature for the all ages and over-65 years age groups, respectively. Overall effects of 1°C increase in daily mean temperature were associated with a 2.7% (95% CI: 1.7–3.7%) increase in all-cause mortality for all ages and 3.4% (95% CI: 2.1–4.8%) increase for over 65 years old across the six cities when same-day models were fitted.

The overall percent increase for CVM as well as city-specific percent changes was the highest in the 1-day lagged model, compared to no-lagged and lagged by 2–5 days models (data not shown). Therefore, we chose the 1-day lagged model and results for CVM in Tables 2, 4 were from 1-day lagged model. The effect of high temperature on CVM varied by city; those for Daejeon and Incheon were especially high, showing 10.7% (95% CI: 0.4–21.9%) and 8.6% (95% CI: 2.5–15.1%) for all ages, respectively, whereas results for the other cities were not statistically significant. Overall effects on CVM were 3.8% (95% CI: 0.8–6.9%) and 4.6% (95% CI: 0.4–8.9%) for all ages and over 65 years old, respectively, showing a greater value than the all-cause mortality data.

### CLIMATE CHANGE AND EXCESS MORTALITY

Table 3 shows the temperature change between present and future and added days with daily mean temperature over *T_q_^c^*, the 75th percentile of present summer temperature by city. For the upper 25% of summer temperature, the averages of daily mean temperature were predicted to increase ranging from 1.9°C (Daejeon under RCP 4.5) to 3.7°C (Daegu under RCP 8.5) in 2041–2070 and from 2.5°C (Daejeon under RCP 4.5) to 5.9°C (Incheon under RCP 8.5) in 2071–2100. For the added days with daily mean temperature over *T_q_^c^*, the averaged increase in temperature ranged from 0.6°C in 2041–2070 (Daegu under RCP 4.5) to 2.9°C in 2071–2100 (Seoul, Incheon and Busan under RCP 8.5). Increases in temperature for the “shifted” days, upper 25% of summertime, were higher than those for the “added” days. Days with daily mean temperature over *T_q_^c^* were predicted to increase by 11–88 days, varying by city and emission scenario.

**FIGURE 3 ∣:**
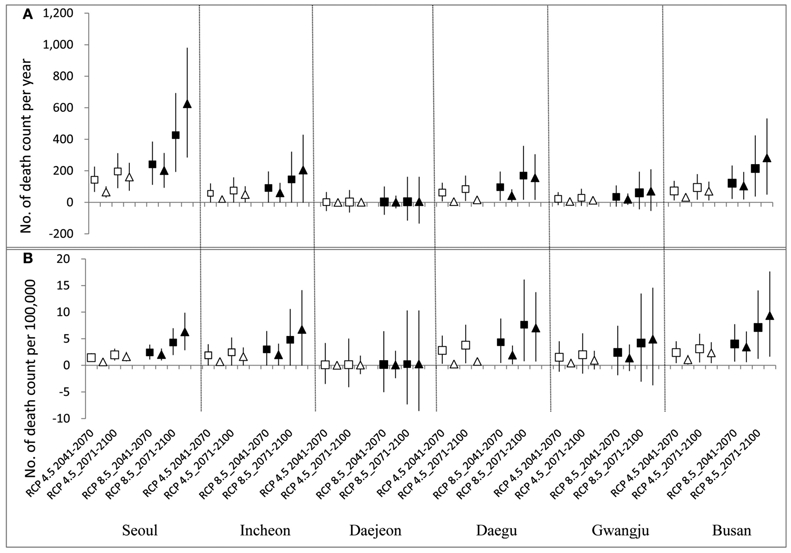
Heat-related mortality attributable to climate change under RCP 4.5 and RCP 8.5. Top graph **(A)** presents absolute values of death count and bottom one **(B)** presents values of death count with adjustment of projected future population scale. (White square and triangle indicate results from “shitted” and “added” effect based on RCP 4.5 scenario and black square and triangle indicate results from “shitted” and “added” effect based on RCP 8.5, respectively; vertical bars indicate 95% confidence intervals).

Figure 3 and Table 4 present city-specific (Figure 3) and overall (Table 4) annual excess mortality due to climate change-induced temperature increase in the future. Estimated excess mortalities for six cities in South Korea were a total of 500 (95% CI: 313–703) deaths per year in 2041–2070 and 766 (95% CI: 478–1077) deaths per year in 2070–2100 based on RCP 4.5 and 1008 (95% CI: 628–1420) deaths per year in 2041–2070 and 2320 (95% CI: 1430–3281) deaths per year in 2070–2100 based on RCP 8.5. Excess CVM was estimated to range from 192 (95% CI: 41–351) to 896 (95% CI: 185–1694) deaths per year, comprising approximately 38.5% of all-cause excess mortality (Comparison of EMs between all-cause mortality and CVM can be seen in Figure S3).

When temperature increase is relatively mild (e.g., 2041–2070 under RCP 4.5), the “added” effects (i.e., the impact induced by increased days with daily mean temperature over *T_q_^c^*) were not higher than the “shifted” effects (i.e., impact induced by temperature increment of upper 25% of summer temperature). However, the “added” effects increased as temperature difference increased. The overall “added” effect for six cities in 2070–2100 under RCP 8.5 was 1273 (95% CI: 784–1775) deaths per year; this is about 22% higher than the “shifted” effect (1047 (95% CI: 646–1506)).

Estimated future heat-related mortality varied by city as shown in Figure 3. Absolute death counts attributable to future temperature increase were the highest in Seoul, showing 1053 (95% CI: 478–1675) deaths per year in 2070–2100 under RCP 8.5 when the “shifted” (427 (95% CI: 193–694) deaths per year) and the “added” effect (626 (95% CI: 284–981) deaths per year) were summed (Figure 3A). However, when the excess death counts were adjusted with the future population scale of each city, those for Busan (17 (95% CI: 3–32)) deaths per 100,000 persons in 2071–2100 under RCP 8.5, located at a relatively lower latitude, were higher than Seoul (11 (95% CI: 5–17)) deaths per 100,000 persons in 2071–2100 under RCP 8.5 (Figure 3B).

## DISCUSSION

We have assessed excess mortality attributable to future climate change in six Korean cities by applying the estimated mortality risks, projected populations, and projected daily mean temperatures based on RCPs. Our findings support the assumption that the impact of future temperature increment on human health will be profound and indicate that we can reduce the health burden of climate change substantially by lowering future carbon dioxide emission.

To directly assess the temperature change between the present and future while considering increased days with over a threshold temperature and shifted hot-to-hotter temperature, we separated future summer days into two categories as follows: days with the upper 25% of daily mean temperature and days with lower 75% of daily mean temperature. This made it possible to evaluate the “shifted” and the “added” temperature effects of future climate change on mortality, as well as to directly compute excess mortality due to climate change-induced temperature increase. The daily mean temperature over the 75th percentile of summertime for the future was expected to be much higher than the present; that is, the averaged daily mean temperature of upper 25% during the summer would be 32.1°C in Seoul and 33.0°C in Daegu in 2071–2100 (Table S1) based on RCP 8.5, which represents the highest GHGs emission among four RCPs, whereas those for present-day (2001–2010) are 26.6°C in Seoul and 27.4°C in Daegu (Moss et al., 2010).

In most cases, excess mortality derived from the “shifted” effect exceeded the “added” effect. However, when temperature increase was very high (e.g., 2070–2100 under RCP 8.5), the “added” effects on mortality were predicted to surpass the “shifted” effects in the future (Table 4 and Figure 3). It is mainly because the number of increased days with daily mean temperature over threshold, the 75th percentile of present summer temperature (*T_q_^c^*), would increase considerably.

Previous studies used similar “health impact function” to this study (Dessai, 2003; Knowlton et al., 2007) For example, Knowlton et al. (2007) first calculated daily heat-related premature deaths attributable to high temperature over thresholds and summed them to estimate annual deaths for both present (1990s) and future (2050s). They then calculated percent change of future premature deaths compared with present premature deaths. When we applied the method presented by Knowlton et al. (2007) for Seoul, Korea, assuming the 75th percentile of temperature as a threshold, heat-related premature deaths for present (2001–2010) were 77 deaths per year and 1158 deaths per year for future (2071–2100) under RCP 8.5. Therefore, the difference, 1081 deaths per year, could be considered as excess mortality due to the climate change-induced temperature increase. This result is very similar to the results of our current study; total excess mortality for Seoul derived from our study was about 1053 deaths per year for 2071–2100 under RCP8.5 when the “shifted” and “added” effects were summed (Figure 3A). We directly estimated excess mortality due to climate change-induced temperature increase by dividing the temperature of future summertime into two temperature levels. We then assessed excess mortalities caused by “shifted” and “added” effects, separately. This approach helps us to make more detailed evaluation of the heat-related premature deaths. Relatively low temperature increase in the future will cause heat-related excess mortality mainly attributable to shifted hot to hotter temperature. In contrast, high temperature increase will cause heat-related excess mortality more attributable to the newly added days above the threshold temperature than the shifted hot days.

Regarding the mortality risk, we fitted GLM using the upper 25% of temperature during present summertime to estimate mortality risks in the six cities. The mortality risk can vary by percentiles of temperature even in a city. A previous study showed that the percent change of mortality risk for the 90th percentile (25°C) to the 50th percentile (15°C) of year round temperature in Seoul during 2000–2007 was 10.2% (95% CI: 7.4–13.0%) and that for the 99th percentile (29°C) to the 90th percentile (25°C) was 3.9% (95% CI: −0.3–8.3%) (Son et al., 2011). Hajat et al. (2006) reported that heat wave effects resulted from linear slope models varied by percentiles of temperature, showing that the higher percentile is, the greater the slope is. Our previous study showed that estimated percent increase for the 90th percentile of temperature during summertime (9.3% (95% CI: −0.4–19.9%)) was 2.7-fold greater than that for the 75th percentile of temperature (3.5% (95% CI: 1.0–6.0%)) in Daegu, South Korea (Kim and Joh, 2006). We selected the 75th percentile of daily mean temperatures of present summertime as a threshold for each city in order to include as many days with high temperatures that cause heat-related excess mortality as possible.

All-cause mortality risks to high temperature in this study were estimated to increase by 2.7% (95% CI: 1.7–3.7%) for all ages and by 3.4% (95% CI: 2.1–4.7%) for over 65 years old across the six cities. The predicted mortality risks of high temperature in this study are quite robust and in accordance with other studies. Our previous study presented that percent change of all-cause mortality caused by daily maximum temperature over 27.9°C was 2.6% (95% CI: 1.5–3.8%) in Seoul when applied by GAM for 2000–2002 (Kim and Joh, 2006). Percent change of all-cause mortality for Seoul in this study is 2.6% (95% CI 1.2–4.5%) for daily mean temperature over 25.5°C (Table 2). Studies from other countries also show similar results (Curriero et al., 2002; Tagaris et al., 2009; Guo et al., 2011). For example, Curriero et al. (2002) observed that mortality rate due to daily mean temperature over 21.5°C had increased by 3.7% in Washington, DC for 1973–1994.

Mortality risks in this study show inconsistency between all-cause and cardiovascular mortality and between cities. For example, the all-cause mortality risk in Seoul is significant while CVM risk is not, whereas in Incheon, CVM risk is quite high with significance while all-cause mortality risk is insignificant. Uncertainty of statistical modeling and differences of these populations are possible causes. The magnitudes of daily death counts vary by city, which is related to the statistical significance. In particular, most death counts of CVM are very small, e.g., those for Daejeon and Gwangju are less than 5, which might cause the nonsignificant RR or wide range of confidence interval for CVM in the cities. The effect of heat stress on mortality varies by latitude, cause of disease, lagged days, and demographic characteristics (Curriero et al., 2002; Hajat and Kosatky, 2010). Besides, Schwartz (2005) reported that patients with diabetes had a higher risk of dying on hot days than other subjects (odds ratio, 1.17; 95% CI, 1.04–1.32), which suggest that all-cause mortality risk due to heat stress be able to be higher than that of CVM. Therefore, it can be inferred that in Seoul, heat stress impacts on mortality not through aggravation of cardiovascular disease but through other diseases such as diabetes, whereas in Incheon, cardiovascular disease patients are more vulnerable to heat stress than other cities. The different responses to heat waves among cities might be caused by their different adaptation capacity including their emergency response systems. However, we did not consider such socioeconomic factors, which is a limitation of our study.

Some studies have reported mortality displacement. Hajat et al. (2005) reported that mortality displacement exists in London, where there is higher proportion of elderly people than other cities. These results might imply that excess daily mortality results from short-term displacement, known as the “harvesting” effect. However, Hajat et al. (2005) also observed no mortality displacement in other European cities, except for London. Basu and Malig (2011) stated that the evidence of mortality displacement was not found since no significant negative effects were observed in the following single or cumulative days in California, USA. Mortality displacement may not be robust and may vary by city. According to a previous study about heat-related mortality in South Korea, cumulative percent increase of mortality over 30 days was still significant and greater than the single-day effect in Seoul and Incheon (Ha et al., 2011). Ha et al. (2011) used a distributed lag model, which considers a cumulative effect that the single-day model cannot identify, and concluded that summer high temperature did not just advance the date of premature deaths by a few days, but its impacts on mortality was robust. Therefore, excess mortality estimated in this study cannot be considered just mortality displacement.

One of the limitations of our analysis is that simulations from only one climate model were used. This is because there are no other archived model projections based on the new emission scenarios, the RCPs, in South Korea. Although the simulated future temperature vary by model and scenario (Peng et al., 2011), the CCIC-KMA results used in this study have showed similar temperature increases to previous studies that had simulated future temperature in South Korea using different climate models and emission scenarios (Boo et al., 2005; Koo et al., 2009). Boo et al. (2005) reported that simulated daily temperature for the period of 2071–2100 was projected to shift by about 5.5°C compared to the period of 1971–2000 in South Korea. The increased temperature resulted in increase in frequency and intensity of hot weather when the temperature was predicted based on the IPCC *Special Report on Emissions Scenario* (SRES) A2, which assumes 830 ppm of CO_2_ in 2100. The temperature change predicted by Boo et al. (2005) is similar to the temperature increase in 2071–2100 based on RCP 8.5. In addition, Koo et al. (2009) reported that regional climate projection using Fifth-Generation Penn State/NCAR Mesoscale Model (MM5) dynamic-downscaling simulation showed that the annual mean value of daily mean temperatures will increase by 3.8°C between the present and 2071–2100 based on the SRES A1B scenario, which assumes 720 ppm of CO_2_ in 2100. These previous studies as well as the CCIC-KMA results have shown consistent temperature increases in South Korea in the future, exceeding the globally averaged temperature increase. Furthermore, according to a previous study on future temperature distribution, changes in the mean and variability of temperature analyzed by temperature projection time series are not influenced by climate model bias (Gosling et al., 2009). Despite of the consistency in temperature increase in South Korea in the future, there exists uncertainty in the projected temperature changes. Without additional model simulations available, our current analysis cannot quantify this uncertainty, nor can it evaluate the effectiveness of any potential policy interventions. Nonetheless, we selected two RCPs, i.e., the most plausible emission pathway (RCP 4.5) and the highest emission pathway (RCP 8.5) as future temperature changes would likely be bounded by these two scenarios. Using ensemble model simulation results will be our future research direction.

We have not estimated the excess mortality in the context of demographic vulnerability to climate change due to a lack of detailed future population data. Further studies on the heat-related excess mortality based on age and gender specific future population and mortality risk are required. Another limitation of this study is not accounting for acclimatization of people to the warming climate. Knowlton et al. (2007) reported that projected regional increases in heat-related premature mortality by the 2050s ranged from 47 to 95% increase compared with the 1990s. This increase was reduced by about 25% when acclimatization including air-conditioning was considered. However, we could not consider this effect because there is no study on the acclimatization in South Korea.

To our knowledge, this study is the first to quantify the health impact of future climate change in South Korea. We applied city-specific mortality risks for six cities to estimate the health impact of temperature increment due to climate change in the future. Furthermore, we suggested a method to directly assess the health impact of increased temperature due to climate change while distinguishing the effects of shifted high to higher temperature and increased days over a temperature threshold. With this approach, we could make more detailed evaluation of the heat-related premature deaths caused by climate change.

## CONCLUSIONS

The association between high temperature and mortality in six South Korean cities is statistically significant, implying that the heat-related mortality may be accelerated by climate change in the future. Estimated impacts of climate change on heat-related mortality based on projected future temperature under RCPs, estimated mortality risks, and projected population will be profound and vary by city. Excess mortality rate due to future climate change is higher in cities located at lower latitudes than those at higher latitudes, while the absolute value of excess mortality is high in city with high population and mortality incidence. The estimated mortality attributable to climate change based on the fossil fuel-intensive emission scenario is much greater than that based on the low to medium emission scenario. This implies that efforts to mitigate climate change can bring about substantial health benefits by reducing the heat related mortality.

## Supplementary Material

Supplement **Figure S1 ∣ Scatter plot of observed and simulated daily mean temperature for six cities in Korea during summertime.** The daily mean temperature was averaged by day of year for 2001–2008.
**Figure S2 ∣ Temperature shift during summertime caused by climate change.** Dotted vertical red line indicates temperature of current 75th percentile of summer temperature (June to September) and solid red line is for future; White bars are frequency of current and gray bars are future daily mean temperature in summer; *T_q_* indicates 75th percentile of temperature during current summer and *T′_q_* is 75th percentile of temperature during future summer; Gray-colored days between *T_q_* and *T′_q_* are newly added days with temperature over 75th percentile of current summer days (*T_q_*) in future; Orange-colored days are days with temperature over 75th percentile of future summer days (*T′_q_*).
**Figure S3 ∣ Sum of heat-related mortality attributable to climate change in six cities, South Korea.** Vertical bars indicate 95% confidence intervals reflecting statistical uncertainty in risk estimation.
Table S1 ∣ Summary of simulated temperature under two RCP^a^ scenarios.

## Figures and Tables

**Table 1 ∣:** Summary statistics of the study population, weather, and pollution variables of six cities in Korea, 2001–2008, summertime (June through September).

City^a^	Population		Daily death count			Temperature^c^(°C)		Relative humidity (%) mean (*SD*)	PM_10_ (μg/m^3^) mean (*SD*)	O_3_^e^ (ppb) mean (*SD*)
	2008	2040	All cause	Cardiovascular	Respiratory	mean (*SD*)	75th^d^			
Seoul	10,081,017	9,924,373	88.5 (10.0)^b^	23.7 (5.0)	4.7 (2.3)	23.6 (2.8)	25.5	70.7 (12.2)	48.7 (28.2)	27.7 (13.9)
Incheon	2,681,825	3,036,476	25.2 (5.2)	7.1 (2.8)	1.5 (1.2)	23.0 (2.6)	24.9	76.6 (11.2)	48.5 (23.7)	29.5 (13.0)
Daejeon	1,497,857	1,566,886	12.9 (3.6)	3.4 (1.8)	0.9 (1.0)	23.6 (2.9)	25.6	73.7 (10.4)	35.3 (17.8)	12.9 (3.5)
Daegu	2,475,410	2,220,439	25.9 (5.2)	6.8 (2.7)	1.5 (1.3)	24.4 (3.4)	27.0	69.6 (11.2)	45.5 (18.5)	32.1 (14.7)
Gwangju	1,462,133	1,437,531	13.4 (3.6)	3.1 (1.7)	0.8 (1.0)	24.1 (2.9)	26.2	74.3 (10.0)	38.8 (20.8)	28.9 (12.8)
Busan	3,506,377	3,014,946	42.7 (6.8)	12.7 (3.6)	2.3 (1.5)	23.8 (2.9)	25.4	77.3 (10.3)	50.6 (19.9)	31.1 (12.0)

a Cities are arranged by latitude; the latitude of Seoul is 37.57° showing the highest latitude and that of Busan is 35.10° showing the lowest latitude.

b Standard deviation.

c Daily mean temperature.

d 75th percentile of summertime, June through September.

e Daily mean ozone.

**Table 2 ∣:** Percent change in mortality risk for 1°C increase in daily mean temperature.

City	Age group	(Unit: %)		
		All-cause mortality	Cardiovascular mortality	Respiratory mortality
Seoul	All ages	2.62 (1.20, 4.05)	1.17 (−1.41, 3.82)	0.51 (−5.28, 6.65)
	Age ≥65 years	3.30 (1.47, 5.17)	0.08 (−2.74, 2.99)	2.55 (−3.85, 9.38)
Incheon	All ages	2.80 (−0.02, 5.70)	8.57 (2.45, 15.06)	5.70 (−5.30, 17.97)
	Age ≥65 years	2.96 (−0.74, 6.80)	12.48 (4.55, 21.01)	0.96 (−10.77, 14.22)
Daejeon	All ages	0.14 (−4.81, 5.35)	10.66 (0.43, 21.92)	3.41 (−16.68, 28.33)
	Age ≥65 years	1.36 (−5.22, 8.39)	11.31 (−1.28, 25.51)	5.75 (−15.72, 32.69)
Daegu	All ages	3.48 (0.37, 6.67)	5.18 (−0.88, 11.60)	0.32 (−12.11, 14.50)
	Age ≥65 years	5.76 (1.62, 10.06)	4.33 (−2.14, 11.23)	−3.73 (−16.55, 11.06)
Gwangju	All ages	2.50 (−1.96, 716)	−2.08 (−11.47, 8.31)	−5.87 (−24.4, 17.20)
	Age ≥65 years	5.72 (−0.23, 12.01)	−1.37 (−12.63, 11.34)	−2.25 (−22.45, 23.20)
Busan	All ages	3.02 (0.54, 5.55)	3.10 (−1.82, 8.26)	6.68 (−4.20, 18.80)
	Age ≥65 years	2.68 (−0.31, 5.76)	4.97 (−0.63, 10.90)	7.52 (−4.31, 20.82)
Overall	All ages	2.70 (1.67, 3.73)	3.81 (0.82, 6.89)	2.06 (−2.19, 6.50)
	Age ≥65 years	3.44 (2.13, 4.76)	4.56 (0.40, 8.89)	2.35 (−2.28, 7.19)

We estimated the high temperature effects on mortality using generalized linear model with natural cubic splines based on same day temperature.

**Table 3 ∣:** Temperature increment and increased days over 75th percentile temperature of current summertime (June through September) due to climate change.

City	RCP^a^ 4.5						RCP 8.5					
	2041–2070			2071–2100			2041–2070			2071–2100		
	Temperature increment (°C)		Added days^d^	Temperature increment (°C)		Added days	Temperature increment (°C)		Added days	Temperature increment (°C)		Added days
	Shifted^b^	Added^c^		Shifted	Added		Shifted	Added		Shifted	Added	
Seoul	2.0	0.8	35	2.6	1.2	54	3.2	1.3	62	5.5	2.9	88
Incheon	2.3	0.7	34	3.1	1.2	49	3.5	1.2	56	5.9	2.9	80
Daejeon	1.9	0.7	16	2.5	0.9	38	3.2	1.3	38	5.4	2.5	73
Daegu	2.5	0.6	11	2.9	0.6	30	3.7	1.5	33	5.6	2.3	75
Gwangju	2.0	1.1	16	2.6	1.0	38	3.1	1.2	44	5.2	2.5	75
Busan	2.2	1.3	19	2.8	1.2	47	3.4	1.6	53	5.3	2.9	76

a RCP, representative concentration pathway.

b “Shifted” indicates increase in averaged daily mean temperature of upper 25% of future summertime compared with present-day.

c “Added” indicates average of increased temperature over the 75th percentile of present summertime for increased days in the future.

d Added days over 75th percentile of present summertime temperature.

**Table 4 ∣:** Excess mortality attributable to climate change-induced temperature increase in six cities, South Korea.

Climate scenario	Year	(Unit: death count per year)		
		Shifted^a^ Mean (95% CI)	Added^b^ Mean (95% CI)	Total Mean (95% CI)
**ALL-CAUSE MORTALITY**				
RCP 4.5	2041–2070	388.1 ( 243.6, 548.7)	111.8 ( 69.5, 154.5)	499.9 ( 313.1, 703.1)
	2071–2100	504.7 ( 315.9, 715.9)	261.0 ( 162.1, 360.0)	765.7 ( 478.0, 1076.8)
RCP 8.5	2041–2070	619.2 ( 386.4, 880.9)	389.1 ( 241.3, 539.0)	1,008.4 ( 627.6, 1,420.0)
	2071–2100	1,046.7 ( 645.9, 1,505.9)	1,272.9 ( 783.9, 1,775.1)	2,319.5 ( 1,429.8, 3,280.9)
**CARDIOVASCULAR MORTALITY**				
RCP 4.5	2041–2070	149.7 ( 31.8, 275.1)	42.1 ( 9.1, 76.0)	191.8 ( 40.9, 351.1)
	2071–2100	195.3 ( 41.1, 362.5)	98.4 ( 21.2, 177.9)	293.7 ( 62.4, 540.4)
RCP 8.5	2041–2070	240.4 ( 50.2, 450.2)	147.0 ( 31.6, 267.1)	387.4 ( 81.7, 717.3)
	2071–2100	411.4 ( 83.1, 796.7)	484.3 ( 102.0, 897.7)	895.7 ( 185.1, 1,694.4)

a “Shifted” effect indicates excess mortality derived from hot days with upper 25% of daily mean temperature during future summer days.

b “Added” effect indicates excess mortality due to added days with daily mean temperature over threshold in the future.
