# Supplementary material for: The impact of climate change on heat-related mortality in six major cities, South Korea, under representative concentration pathways (RCPs)
Source: Front Environ Sci. Author manuscript; Available in PMC 2021 Jun 15. (PMC8204571; doi:10.3389/fenvs.2014.00003)
Supplement: Supplement — Figure S1 ∣ Scatter plot of observed and simulated daily mean temperature for six cities in Korea during summertime. The daily mean temperature was averaged by day of year for 2001–2008. Figure S2 ∣ Temperature shift during summertime caused by climate change. Dotted vertical red line indicates temperature of current 75th percentile of summer temperature (June to September) and solid red line is for future; White bars are frequency of current and gray bars are future daily mean temperature in summer; Tq indicates 75th percentile of temperature during current summer and T′q is 75th percentile of temperature during future summer; Gray-colored days between Tq and T′q are newly added days with temperature over 75th percentile of current summer days (Tq) in future; Orange-colored days are days with temperature over 75th percentile of future summer days (T′q). Figure S3 ∣ Sum of heat-related mortality attributable to climate change in six cities, South Korea. Vertical bars indicate 95% confidence intervals reflecting statistical uncertainty in risk estimation. Table S1 ∣ Summary of simulated temperature under two RCPa scenarios. [file NIHMS1702309-supplement-Supplement.docx]

**Supplementary material**

**
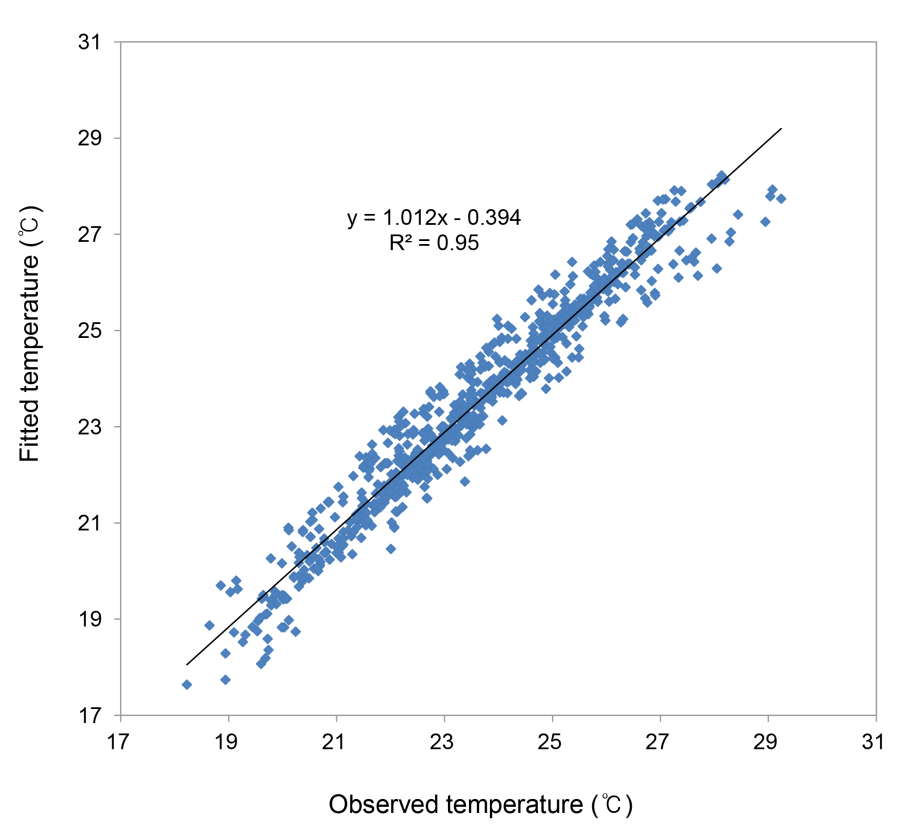
**

**Figure S1. Scatter plot of observed and simulated daily mean temperature for six cities in Korea during summertime.** The daily mean temperature was averaged by day of year for 2001-2008.


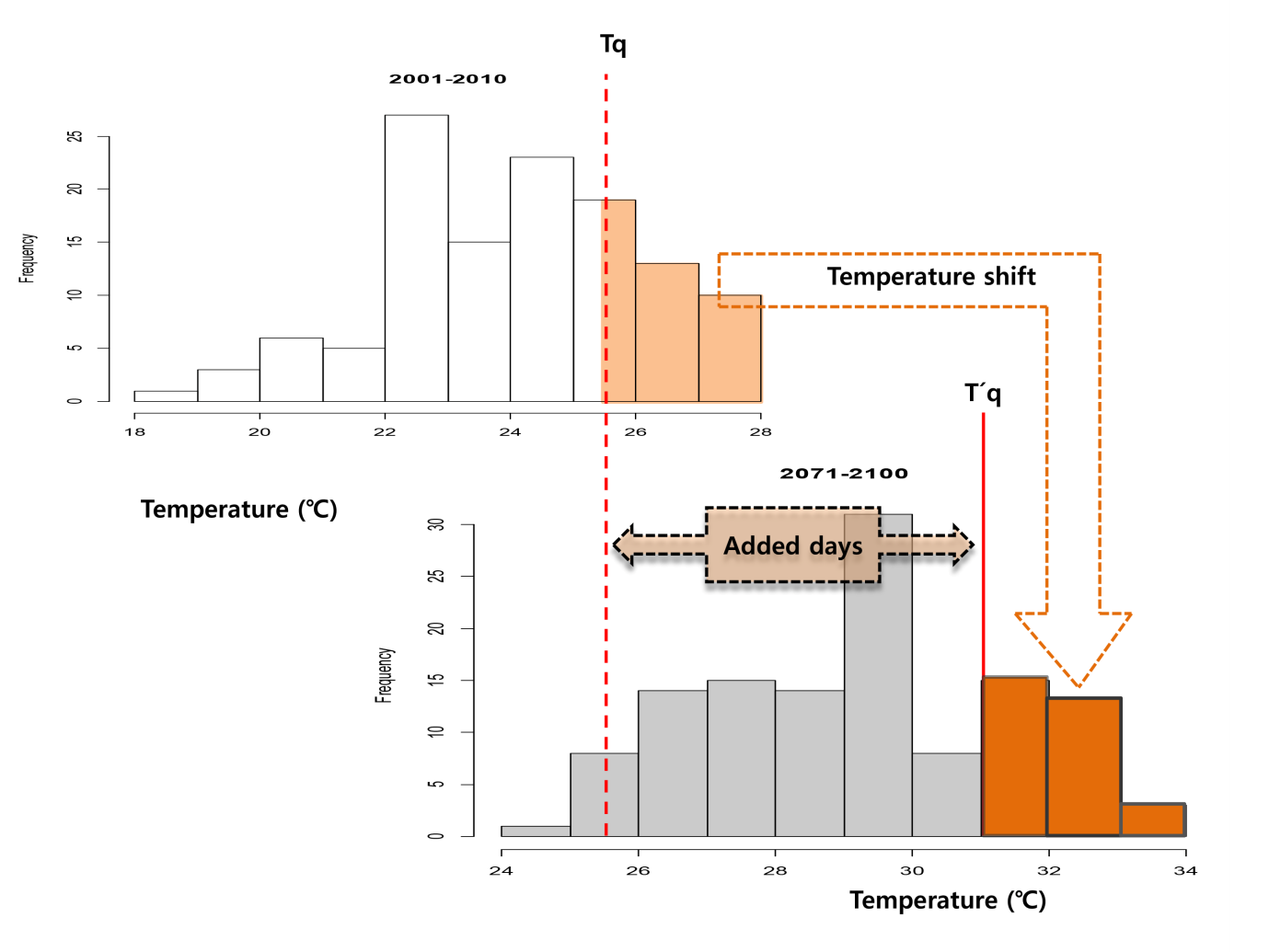


**Figure S2. Temperature shift during summertime caused by climate change.**

Dotted vertical red line indicates temperature of current 75^th^ percentile of summer temperature (June to September) and solid red line is for future; White bars are frequency of current and gray bars are future daily mean temperature in summer; T_q_ indicates 75^th^ percentile of temperature during current summer and T^´^_q_ is 75^th^ percentile of temperature during future summer; Gray-colored days between T_q_ and T^´^_q_ are newly added days with temperature over 75^th^ percentile of current summer days (T_q_) in future; Orange-colored days are days with temperature over 75^th^ percentile of future summer days (T^´^_q_).


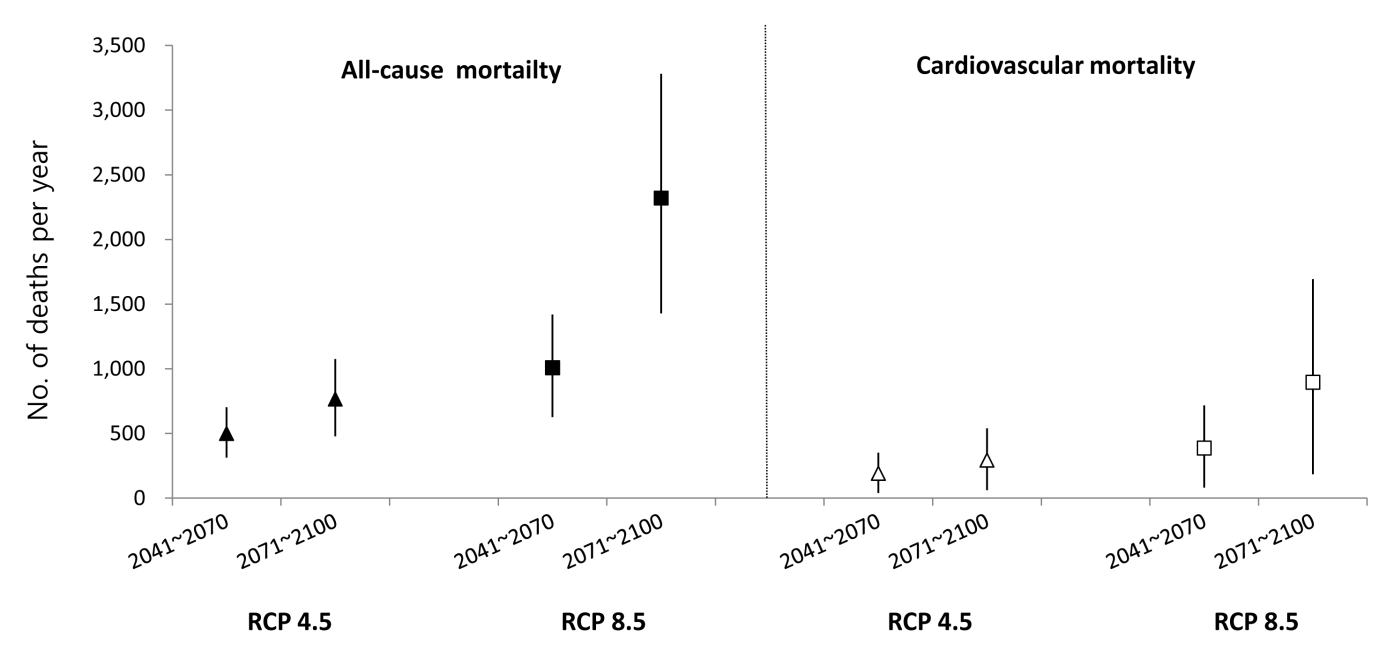


**Figure S3. Sum of heat-related mortality attributable to climate change in six cities, South Korea.**

Vertical bars indicate 95% confidence intervals reflecting statistical uncertainty in risk estimation.

Table S1. Summary of simulated temperature under two RCP^a^ scenarios.

| City | Present-day | RCP4.5 | | RCP8.5 | |
| --- | --- | --- | --- | --- | --- |
|  | 2001-2010  Mean (°C) | 2040s  Mean (°C) | 2070s  Mean (°C) | 2040s  Mean (°C) | 2070s  Mean (°C) |
| Upper 25%^b^ |  |  |  |  |  |
| Seoul | 26.6 | 28.5 | 29.2 | 29.8 | 32.1 |
| Incheon | 25.5 | 27.8 | 28.5 | 29.0 | 31.4 |
| Daejeon | 26.0 | 27.9 | 28.4 | 29.1 | 31.4 |
| Daegu | 27.4 | 29.9 | 30.4 | 31.1 | 33.0 |
| Gwangju | 26.8 | 28.7 | 29.3 | 29.9 | 32.0 |
| Busan | 26.3 | 28.5 | 29.1 | 29.7 | 31.6 |
| Lower 75%^c^ |  |  |  |  |  |
| Seoul | 23.2 | 24.8 | 25.7 | 26.0 | 28.3 |
| Incheon | 21.9 | 23.9 | 24.7 | 24.9 | 27.2 |
| Daejeon | 22.4 | 23.9 | 24.7 | 25.2 | 27.5 |
| Daegu | 23.4 | 24.9 | 25.7 | 26.4 | 28.7 |
| Gwangju | 23.2 | 24.6 | 25.4 | 25.9 | 28.0 |
| Busan | 22.6 | 24.2 | 25.1 | 25.6 | 27.7 |

a) RCP: representative concentration pathway;

b) Upper 25% of daily mean temperature during summertime;

c) Lower75% of daily mean temperature during summertime.
